# Curcumin Nanoparticles-related Non-invasive Tumor Therapy, and Cardiotoxicity Relieve

**DOI:** 10.2174/0109298673305616240610153554

**Published:** 2024-06-24

**Authors:** Yuhang Cheng, Qian Xu, Miao Yu, Chenwei Dang, Limei Deng, Huijun Chen

**Affiliations:** 1 Department of Cardiology, Heilongjiang University of Traditional Chinese Medicine, Harbin, 150006, Heilongjiang, China;; 2 Department of Cardiology, Second Affiliated Hospital of Heilongjiang University of Traditional Chinese Medicine, No. 411, Guogeli Road, Nangang District, Harbin, Heilongjiang Province, 150081, China

**Keywords:** Curcumin, curcumin nanoparticles, cardiotoxicity, tumor therapy, radiotherapy, chemotherapy

## Abstract

Non-invasive antitumor therapy can treat tumor patients who cannot tolerate surgery or are unsuitable. However, tumor resistance to non-invasive antitumor therapy and cardiotoxicity caused by treatment seriously affect the quality of life and prognosis of patients. As a kind of polyphenol extracted from herbs, curcumin has many pharmacological effects, such as anti-inflammation, antioxidation, antitumor, **etc*.* Curcumin plays the antitumor effect by directly promoting tumor cell death and reducing tumor cells' invasive ability. Curcumin exerts the therapeutic effect mainly by inhibiting the nuclear factor-κB (NF-κB) signal pathway, inhibiting the production of cyclooxygenase-2 (COX-2), promoting the expression of caspase-9, and directly inducing reactive oxygen species (ROS) production in tumor cells. Curcumin nanoparticles can solve curcumin's shortcomings, such as poor water solubility and high metabolic rate, and can be effectively used in antitumor therapy. Curcumin nanoparticles can improve the prognosis and quality of life of tumor patients by using as adjuvants to enhance the sensitivity of tumors to non-invasive therapy and reduce the side effects, especially cardiotoxicity. In this paper, we collect and analyze the literature of relevant databases. It is pointed out that future research on curcumin tends to alleviate the adverse reactions caused by treatment, which is of more significance to tumor patients.

## 1 INTRODUCTION

At present, tumors are the most threatening disease to human health globally [1]. Its characteristics include high mortality, poor prognosis, and so on. Due to the complexity of the tumor growth process and individual differences in tumorigenesis, there are many differences in tumor treatment. Tumor therapy methods mainly include surgery, radiotherapy (RT), chemotherapy, immune therapy, photodynamic therapy (PDT), and sonodynamic therapy (SDT) [2]. The surgery is traumatic to the human body. At the same time, some patients cannot tolerate the surgery or are unsuitable for surgical treatment, so other non-invasive tumor treatment methods have attracted people's attention. For example, patients with head and neck tumors that are unsuitable for surgery are mostly treated with RT, chemotherapy, or other treatments [3, 4]. However, there still are some limitations in tumor non-invasive treatments, such as radiation tolerance, chemotherapy resistance, chemotherapy drug toxicity, and other side effects for humans [5-7].

Curcumin, a polyphenolic compound extracted from herbaceous turmeric, has promising anti-inflammatory and antitumor effects. Because it is extracted from food, curcumin and its homologous compound have minimal side effects on the human body [8]. In the field of tumor treatment, there is a lot of research on curcumin. Curcumin can be used as a chemotherapeutic or adjuvant tumor therapy drug [9, 10]. Curcumin can also be used as a photosensitizer or sonosensitizer for PDT or SDT antitumor therapy [11, 12]. At the same time, curcumin can have an anti-inflammatory effect by alleviating the damage to normal tissue caused by RT [13]. It can also mediate the changes in the tumor immune microenvironment (TIME) and promote antitumor biotherapy by regulating microRNA (miRNA) [14, 15].

Cardiovascular problems, like tumors, are the leading cause of threats to human health and safety [16]. In the process of tumor therapy, whether it is RT, chemotherapy, PDT, SDT, or biotherapy, the potential biological toxicity in the treatment cannot be ignored. Among them, the cardiotoxicity caused by treatment is significant [17]. Whether short-term or long-term cardiotoxicity, it will affect the quality of life of tumor patients and even their survival rate. Therefore, alleviating the cardiotoxicity caused by antitumor therapy is the focus of basic research and clinical trials of antitumor therapy [18].

Previous studies have shown that curcumin could combat cardiotoxicity caused by RT and chemotherapy and alleviate tumor drug resistance. At the same time, combined with curcumin could improve the quality of life of patients [19-21]. However, the disadvantages of curcumin in the human body, such as poor water solubility, low absorption rate, short circulation time, and fast metabolic rate, limit the clinical application of curcumin [22]. For these reasons, the researchers designed drug-loaded structures such as liposomes, nanoparticles, and colloidal bundles with curcumin as the core to solve these problems and improve the effective therapeutic concentration of curcumin in the treatment process [23-25]. Among them, curcumin nanoparticles have higher research value because of their more vigorous passive tumor-targeting ability, smaller particle size, higher stability, better biocompatibility, higher controllability, and other advantages [26, 27].

**Fig. (1):**
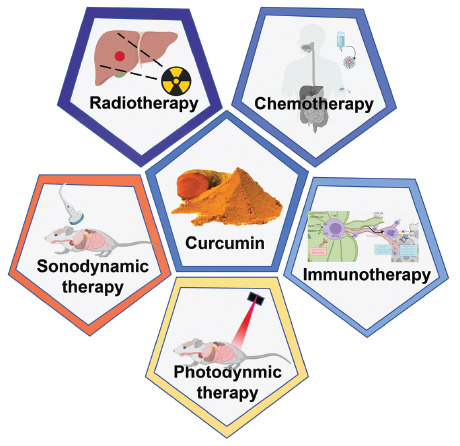
Curcum can be used as an auxiliary or direct means of non-invasive therapy to achieve anti-tumor treatment.

Here, we systematically describe the pharmacological action, mechanism, and nanoparticles of curcumin. At the same time, we give a comprehensive and detailed description of curcumin-related non-invasive tumor therapy, including curcumin-mediated tumor therapy and curcumin as an adjuvant drug to improve the therapeutic effect of antitumor treatment. The mechanism of curcumin synergistic therapy and its cardioprotective effect on non-invasive antitumor therapy were emphasized. We hope the article can guide the curcumin nanoparticles to assist non-invasive tumor therapy in clinical, reduce the side effects of antitumor treatment, and improve the therapeutic effect of non-invasive antitumor methods (Fig. **1**).

## 2 METHODS

We conducted literature searches on PubMed, Embase, and Web of Science from the date of establishment to January 1, 2024. We searched the English literature for curcumin, tumor, antitumor therapy, non-invasive antitumor therapy, adverse reactions related to antitumor therapy, and so on. To ensure the authenticity of the content, we only read and summarized the articles. We determined that the theme of curcumin on cardiotoxicity caused by non-invasive antitumor therapy is of more advanced significance.

## 3 THE PHARMACOLOGY AND ACTION MECHANISM OF CURCUMIN AND THE SIGNIFICANCE OF CURCUMIN NANOPARTICLES

### 3.1 Physicochemical and Metabolic Kinetic Characteristics of Curcumin

**Fig. (2):**
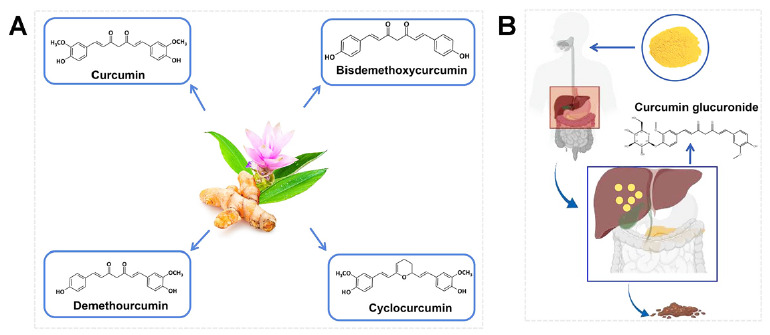
(**A**) The main compound forms of curcumin. (**B**) The intake mode and metabolic pathway of curcumin.

According to the position of a methoxy group(s) in the aromatic ring, curcuminoids can be mainly divided into four categories: curcumin, demethoxy curcumin, bis-demethoxycurcumin, and cyclo curcumin. Among them, curcumin accounts for about 77% (Fig. **2A**) [28, 29]. The chemical formula and chemical name of curcumin (diferuloylmethane) are C_21_H_20_O_6_ and 1,7-bis-(4-hydroxy3-methoxyphenyl)-1,6-heptadieno-3,5-dione respectively, and its molecular weight is 368.38 [30]. Curcumin is mainly extracted from the rhizome of turmeric, a perennial plant in the Curcuma (Zingiberaceae). Curcumin is orange in appearance, slightly bitter in taste, and insoluble in water [31]. The particular chemical structure of curcumin determines its antioxidant activity, and the main mechanisms include phenoxy formation and hydrogen abstraction [32]. Curcumin is composed of a seven-carbon ketone-enol bond connecting two phenyl rings substituted by hydroxyl and methoxy groups. This particular diketone structure is the basis of curcumin's pharmacological action, and its hydroxyl residues are extremely vulnerable to free radicals [33-35]. Curcumin can be administered by oral administration, intravenous injection, intratumoral injection, intracavitary administration, and so on, but its bioavailability is low. The primary metabolites of curcumin are curcumin glucuronide, followed by curcumin sulfate, hexahydrocurcumin, and other chemicals. In mammals, it is metabolized through the liver and mainly excreted through feces and a small amount of urine (Fig. **2B**) [36, 37].

### 3.2 Function and Mechanism of Curcumin

**Fig. (3):**
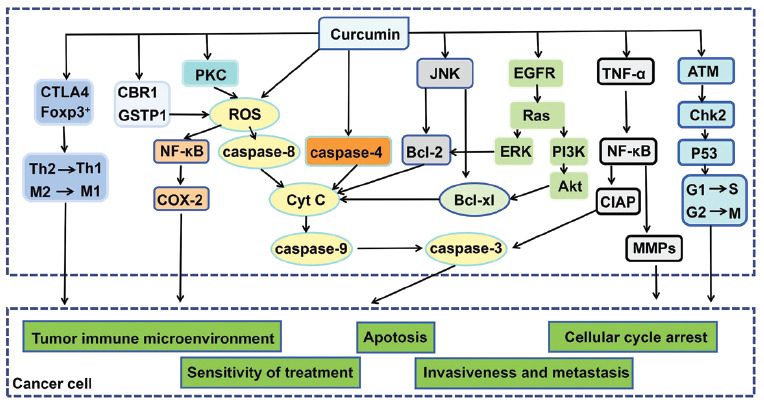
The main anti-tumor signal pathway of curcumin.

The pharmacological effects of curcumin include anti-inflammatory, antioxidant, antitumor, **etc*.* Curcumin exerts its anti-inflammatory effect primarily by inhibiting mitogen-activated protein kinase (MAPK), nuclear factor-κB (NF-κB) and other signal pathways, down-regulating the activities of cyclooxygenase-2 (COX-2), lipoxygenase (LOX), inducible nitric oxide synthase (iNOS) and so on, reducing the production of proinflammatory cytokines such as IL-2, tumor necrotic factor-α (TNF-α), monocyte chemoattractant protein-1 and inflammatory chemokines [38-41]. The particular chemical structure of curcumin can form phenoxy through a series of processes such as electron conversion and proton loss and attract free electrons to attack hydroxyl residues to play its antioxidant role [42-44]. In the process of antitumor therapy, on the one hand, curcumin can directly induce tumor cell apoptosis and block the cell cycle to play an antitumor effect directly [45-47]. Curcumin can induce lethal levels of reactive oxygen species (ROS) in tumors and inhibit the activity of many enzymes involved in ROS metabolism (carboxyl reductase 1 (CBR1), Glutathione-S-transferase phi 1 (GSTP1), **etc*.*), promote the accumulation of ROS in tumor cells, and activate ROS-mediated mitochondrial-related apoptosis in tumor cells [48-50]. Besides, curcumin can promote tumor cell apoptosis by activating intracellular caspase-9 and caspase-3, reducing the expression of p53, inhibiting Bcl2, and promoting the expression of Bax and down-regulating the proportion of Bcl2/Bax [51-53]. Moreover, curcumin can also promote apoptosis by activating caspase-4 and stimulating the Endoplasmic reticulum (ER) stress pathway and mitochondria stress pathway in tumor cells [54]. Curcumin acts on the ATM/Chk2/P53 signaling pathway. It reduces the expression of cyclin D1, cyclin kinase-dependent kinase 2 (CDK2), cdc2/cyclin B complex, and other cell cycle-related proteins, and blocks tumor cells from G1 / S phase and G2 / M phase, thus exerting an antitumor effect [55-58]. On the other hand, curcumin inhibits tumor invasion and metastasis by inhibiting NF-κB and other signaling pathways, such as chemokine and matrix metalloproteinases (MMPs) [59, 60]. Curcumin can regulate the proportion of immune cells in tumors by inhibiting the expression of Foxp3 and other cytokines, restoring the expression of effector T cells (CD8^+^), promoting the transformation of Th2 cytokines to Th1 cytokines, inhibiting T cell apoptosis, reducing the expression of Treg cells, reversing tumor immune escape and boosting systemic antitumor immunity (Fig. **3**) [61-65].

### 3.3 Curcumin Nanoparticles

Curcumin has various pharmacological effects, and its good antitumor effect and high biological safety determine its essential position in antitumor therapy. However, the poor water solubility, low absorption, fast metabolic rate of curcumin, and other disadvantages seriously limit its application in antitumor treatment [66]. Although curcumin preparations such as liposomes and colloids can somewhat solve these problems, they still have their limitations. Some curcumin products have the characteristics of poor tumor-targeting, low stability *in vivo*, easy to be swallowed by macrophages, and so on [67]. Curcumin nanoparticles are mainly composed of curcumin loaded with nanoparticles, which can specifically design the function of the nanocarrier according to the needs of therapy so that it has higher stability, tumor-targeting, and other advantages. For example, ph-responsive curcumin nanoparticles, magnetically driven curcumin nanoparticles, and temperature-responsive curcumin nanoparticles can effectively deliver curcumin to the tumor site and play an antitumor effect [68-70]. These curcumin nanoparticles' design can solve tumor-targeting, tumor internal enrichment, and bioavailability of curcumin. At the same time, the creation of multifunctional curcumin nanoparticles can also achieve the synergistic antitumor effect of curcumin combined with other therapeutic methods, such as SDT antitumor mediated by multifunctional curcumin nanoparticles [71]. The application of curcumin nanoparticles can effectively overcome the shortcomings of curcumin and improve the antitumor effect and synergistic antitumor effect.

## 4 CURCUMIN COMBINED WITH THE NON-INVASIVE ANTITUMOR METHOD AND ITS EFFECT ON ALLEVIATING CARDIOTOXICITY

### 4.1 Curcumin-mediated Radiosensitization and Relief of Side Effects of RT

#### 4.1.1 Curcumin Increases Radiosensitivity of Tumor

RT is a kind of tumor therapy that uses a certain dose of radiation to ionize the tumor to narrow or even eradicate the tumor [72]. Because of the good adjuvant and radical effect of radiotherapy for some specific types of tumors, RT has become one of the critical means of clinical treatment of tumors [73]. The main target of RT is the DNA of tumor cells. DNA exposed to ionizing radiation mainly occurs single bond breakage and double-bond breakage. When DNA is damaged, tumor cells will find and repair the damaged DNA. The therapeutic mechanism of RT lies in the balance between the level of DNA damage caused by radiation on tumor cells and the level of tumor repair of damaged DNA. DNA, which cannot be repaired or repaired normally, leads to the death of tumor cells [74, 75]. The therapeutic effect of RT is affected by radiation dose and tumor sensitivity to radiotherapy. Some tumors are insensitive to RT, and some people, such as children, cannot exposed to too much radiation [76]. Therefore, some radiosensitizers, such as cisplatin, are used in adjuvant radiotherapy [77]. Radiation sensitizers can reduce the dose of radiation, improve the sensitivity of the tumor to RT, and reduce the degree of radiation damage to normal human tissue. The inhibition of NF-κB signal pathway is the primary mechanism of curcumin. Curcumin-assisted RT can effectively inhibit the repair ability of tumor cells to damage DNA and increase the sensitivity of tumor cells to radiation. This sensitization effect has been confirmed in bladder cancer, liver cancer, and other tumors [78-80]. Circular RNA (circRNA) can inhibit miRNA related to tumor growth, invasion, and other functions and participate in the pathological process of tumor growth. Curcumin can regulate the related circRNA and then restrict the circRNA-miRNA-mRNA network, inhibit the ability of tumor cells and cancer stem cells (CSCs) to repair damaged DNA, and achieve radiosensitization of tumor cells [81]. In addition, curcumin can enhance the sensitivity of RT by regulating the expression of cytokines such as epidermal growth factor receptor (EGFR), antioxidant enzyme thioredoxin reductase-1 (TxnRd1), signal transducer and transcriptional activator 3 (STAT3) [82-84]. Curcumin nanoparticles with more functions, such as hemoglobin-curcumin nanoparticles, can target liver cancer tissue to achieve curcumin adjuvant chemotherapy sensitization while loading hemoglobin to alleviate tumor hypoxia microenvironment, reduce the chemotherapy resistance of some tumor cells caused by hypoxia tumor microenvironment (TME), and achieve the effect of synergistic radiosensitization [85].

#### 4.1.2 Protective Effect of Curcumin on Side Effects of RT

RT can damage normal human tissue while treating tumors, and these injuries are called side effects of RT. The side effects of RT are mainly dermatitis, radiation pneumonia, cataracts, cardiotoxicity, and other adverse reactions, which are essentially inflammatory [86-89]. Curcumin has good antioxidant and anti-inflammatory effects. Curcumin can effectively treat and prevent radiation adverse reactions such as radiation dermatitis and radiation pneumonia by reducing the expression of inflammatory factors such as fibrotic cytokines, TNF-α, and IL-1, inhibiting NF-κB signal pathway and reducing oxidative stress [90-92]. The main manifestations of heart diseases caused by RT are pericarditis, aortic root and valvular aortic stenosis and mitral stenosis, congestive heart failure, coronary heart disease, myocardial atrophy, myocardial infarction, pericardial adhesion, and so on [93-95]. Radiation-related cardiotoxicity is mainly caused by the degree of cell death, injury repair, and inflammatory pathway change in the cardiac vascular system during RT [96, 97]. Whether acute or chronic, heart problems caused by RT will become one of the main reasons affecting the quality of life and duration of RT patients [98]. Although there are no specific studies on curcumin alleviating cardiotoxicity induced by RT, its good anti-inflammatory and antioxidant effects can effectively ameliorate cardiotoxicity [99, 100]. Curcumin is expected to be an effective adjuvant in treating cardiotoxicity caused by RT in the future by inhibiting the NF-κB signal pathway, antioxidative stress, and reducing fibrotic cytokines.

### 4.2 Curcumin Alleviates Drug Resistance and Side Effects of Chemotherapy

#### 4.2.1 Curcumin Assists in Relieving Chemotherapy Resistance

Chemotherapy mediates tumor cell death using cytotoxic drugs, which are widely used in clinical treatment [101]. Chemotherapy resistance results in tumor cells gradually evolving the therapeutic effect of anti-chemotherapeutic medications in the complex growth process [102]. Tumor cells can achieve chemotherapeutic drug resistance by promoting drug efflux, antagonizing drug-mediated apoptosis, repairing damaged DNA, changing drug targets, regulating miRNA, *etc* [103-107]. Chemotherapy resistance is a solemn adverse event in the process of chemotherapy. Chemotherapy resistance will reduce the antitumor effect of drugs, thus forcing doctors to increase the concentration of chemotherapy drugs and increase the side effects of chemotherapy drugs on patients and other adverse events. Other treatments to alleviate chemotherapy resistance are standard solutions in clinical therapy and basic research [108, 109]. Previous studies have found that curcumin can effectively help the problem of chemotherapy resistance of tumors, and it can be combined with a variety of chemotherapeutic drugs to solve the problem of drug resistance of many kinds of tumors. Curcumin can reduce the level of ATP binding cassette (ABC) drug transporter in tumor cells, reduce the efflux of doxorubicin, and improve the therapeutic effect of doxorubicin on Hodgkin's lymphoma [110]. In the study of curcumin combined with cisplatin against bladder cancer, curcumin showed a solid synergistic effect by activating caspase-3 and up-regulating the signal transduction of phosphorylated mitogen-activated protein kinase (p-MEK) and phosphorylated extracellular signal-regulated kinase 1 and 2 (p-ERK1/2) [111]. Curcumin can down-regulate the expression of COX-2 and NF-κB in gastric cancer cells, regulate inflammatory factors, and increase the sensitivity of tumor cells to 5-fluorouracil (5-FU) [112]. In addition, curcumin can effectively alleviate the drug resistance of docetaxel, oxaliplatin, metformin, celecoxib, and other chemotherapeutic drugs in treating tumors (Table **1**) [113-116]. These are related to the pharmacological effects of curcumin, such as inducing apoptosis, acting on CSCs, reducing the expression of VEGF, and inhibiting the E-cadherin signal pathway [117-119]. The problem of tumor drug resistance widely exists in clinical tumor treatment. Tumor drug resistance not only limits the choice of treatment but also affects patients' prognosis and quality of life. The rational use of chemotherapeutic sensitizers and alleviating the problem of chemotherapy resistance is helpful in improving the effect of antitumor treatment. As a low-toxic drug, curcumin can effectively help the problem of drug resistance in chemotherapy, so it should be widely used in clinics.

#### 4.2.2 Protective Effect of Curcumin on Side Effects of Chemotherapy

**Fig. (4):**
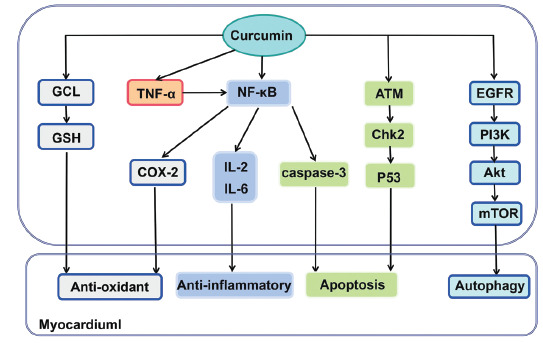
The signal pathway of curcumin in alleviating cardiotoxicity.

Chemotherapeutic drugs mainly mediate tumor cell death in a variety of ways. However, there is also much damage to normal cells caused by chemotherapeutic drugs in the process of tumor treatment. Human treatment mainly shows damage to many organs, such as cardiotoxicity, hepatotoxicity, nephrotoxicity, and so on [120-122]. Curcumin can alleviate cardiotoxicity by regulating myocardial calcium flow, relieving oxidative stress, repairing damaged myocardial DNA, *etc* [123]. At the same time, curcumin alleviates the hepatotoxicity caused by chemotherapy through anti-inflammation and antioxidation, reducing the level of liver fibrosis and blood lipids [124]. Curcumin can also be used to reduce the expression of creatinine and urea nitrogen to alleviate nephrotoxicity [125]. Among the side effects of chemotherapy-related organs, the most significant impact on patients is chemotherapy-related cardiotoxicity. Adverse events such as left ventricular dysfunction, heart failure, coronary heart disease, and thrombus caused by treatment will affect the prognosis and quality of life of patients receiving chemotherapy. Many chemotherapeutic drugs, such as anthracyclines, molecularly targeted drugs, have cardiotoxicity [126-128]. They induce cardiomyocyte death by stimulating oxidative stress, up-regulation of p53 expression, inhibiting the PI3K/AKT/mTOR signal pathway, inhibiting the human epidermal growth factor-2, down-regulation of autophagy-related factors, *etc* [129, 130]. Curcumin inhibits the oxidative stress and apoptosis of cardiomyocytes by inhibiting the NF-κB pathway, down-regulating the expression of COX-2, reducing the levels of inflammatory factors such as TNF- α and IL-2, reducing the activity of caspase-3 and regulating the activities of antioxidant enzymes, to alleviate the cardiotoxicity induced by chemotherapeutic drugs [131-133]. Existing studies have shown that curcumin can increase the level of GSH and protect cardiomyocytes against oxidative damage by inducing glutamate- cysteine ligase (GCL) gene expression [134]. Curcumin inhibits cardiomyocyte apoptosis and other death modes by inhibiting the activity of caspase-3 and other cytokines, such as caspase-1, thus alleviating the injury of cardiomyocytes caused by chemotherapy [135]. Curcumin can also inhibit the chemotaxis of inflammatory cells and reduce the inflammatory reaction of cardiomyocytes and scar formation by inhibiting the NF-κB pathway, down-regulating the expression of COX-2, and reducing inflammatory cytokines such as TNF-α and IL-6 (Fig. **4**) [136]. The protective effect of curcumin increases with the increase of drug concentration in a particular concentration. Curcumin nanoparticles can promote the bioavailability of curcumin, reduce its attention, and make it play a better protective role. The cardiotoxicity of chemotherapy is not only the most important side effect of tumor treatment but also the evaluation index of chemotherapy. Using curcumin to reduce the cardiotoxicity of chemotherapy can improve the therapeutic effect of tumors and patients' prognosis and quality of life.

### 4.3 The Auxiliary Effect of Curcumin on Immunotherapy

#### 4.3.1 Curcumin Enhances the Therapeutic Effect of Immunotherapy

The immune state of the human body changes with the occurrence and development of the tumor, which can be divided into three stages: the “elimination stage,” “balance stage,” and “escape stage.” In the elimination stage, which is in the early stage of tumorigenesis, the human immune system can find tumor cells and initiate the immune response. Most of the tumor cells are killed at this stage. However, with the development of the tumor, the immune system gradually forms a balance with the tumor growth and then enters the balance stage. After the balance stage was maintained for a while, due to the gradual change of the tumor growth environment, the tumor gained the ability to escape from the control of the immune system and entered the escape stage. At this time, the TME is in a state of immunosuppression, and tumor cells can get rid of the monitoring and restriction of the immune system, which leads to tumor growth and metastasis [137, 138]. For example, programmed cell death protein ligand 1 (PD-L1) expressed on the surface of tumor cells can bind to programmed cell death protein 1 (PD-1) expressed on T cells, thus inhibiting the antitumor function of T cells. Activated T cells can express cytotoxic T lymphocyte-associated protein 4 (CTLA-4), a negative regulator of T cell activation, resulting in the inhibition of T cell activation and the inhibition of the immune system [139]. Immunotherapy eliminates tumor patients' immune tolerance by blocking immune checkpoints. Immunotherapy mainly includes passive immunotherapy and active immunotherapy. Passive immunotherapy mainly includes immune checkpoint inhibitors (ICIs), immune checkpoint blocking therapy (ICB), engineering T cells, **etc*.* Active immunotherapy especially involves tumor vaccines. Among them, ICIs are widely used in antitumor therapy. ICIs mainly include monoclonal antibodies, such as ipilimumab for CTLA-4 and nivolumab for PD-1, which can effectively treat tumors [140, 141]. The TIME in the immunosuppressive state will reduce the therapeutic effect of immunotherapy. The TIME mainly comprises regulatory T cells (Tregs), myeloid-derived suppressor cells (MDSCs), M2 macrophages, and other immunosuppressive cells. In the TIME, NK cells, cytotoxic T cells (CTLs), M1 macrophages, and other antitumor immune cells are at a low level [142]. Curcumin can enhance immunotherapy and change the inhibitory state of TIME mainly by inhibiting the transformation of tumor-associated macrophages to M2 macrophages, promoting the transformation of M2 macrophages to M1 macrophages, down-regulating the expression of Tregs and promoting the expression of CD8^+^T cells, and promoting the activation of NK cells [143, 144]. Previous studies have shown that curcumin can inhibit the secretion of cytokines, including IL-6 secreted by MDSCs and IL-2 secreted by Tregs, reduce the expression of Foxp3, and enhance the antitumor effect of CD8^+^T cells [145]. Curcumin extract combined with bevacizumab showed a positive synergistic antitumor effect and better biosafety [146]. The low toxicity and suitable enhanced immune effect of curcumin can be effectively used as an adjuvant of immunotherapy to improve the antitumor effect of immunotherapy. At the same time, curcumin nanoparticles can not only overcome the shortcomings of curcumin but also carry ICIs and improve the safety of immunotherapy, which is the future research direction of curcumin in clinical adjuvant therapy.

#### 4.3.2 Curcumin as a Potential for Alleviating Cardiotoxicity in Immunotherapy

Although immunotherapy plays an antitumor effect mainly by targeting tumor cells, the cardiotoxicity caused by immunotherapy still exists in clinical therapy. There are some adverse cardiac reactions in immunotherapy, such as myocarditis, pericardial disease, left ventricular dysfunction, myocardial infarction, and so on [147]. These heart problems caused by immunotherapy affect the medication regimen, quality of life, and prognosis of patients. There are many manifestations of myocarditis caused by ICIs treatment. Cardiogenic shock and even multiple organ failure can be caused in severe cases. The incidence of ICIs-associated myocarditis is not low. According to statistics, in the process of ICIs treatment, the incidence of myocarditis can be as high as 1.14%. The mechanism of ICIs-associated myocarditis is not clear at present [148]. However, preclinical studies have shown that PD-1-deficient or CTLA-4-deficient mice always suffer from myocarditis, so it may be associated with T cells targeting antigens that express both in the tumor and heart [149]. Similarly, ICIs-related myocardial infarction may be related to the activation of inflammation during immunotherapy, which may lead to the rupture of the fibrous cap of atherosclerotic plaques, resulting in myocardial infarction [150]. We can find that ICIs-related cardiotoxicity is mainly related to inflammation in the heart cells. Curcumin plays different roles in normal cells and tumor cells. Studies have shown that curcumin analog ASC-J9 can alleviate autoimmune myocarditis by reducing the infiltration of M1 and inflammatory cells in mouse cardiomyocytes. Curcumin itself can also relieve autoimmune myocarditis [151, 152]. Although no studies show that curcumin can alleviate the cardiotoxicity caused by immunotherapy at present, it can be speculated that curcumin can reduce the cardiotoxicity caused by immunotherapy-mediated inflammation. This is also a future research direction of curcumin-assisted immunotherapy against tumors.

### 4.4 Curcumin Mediates the Antitumor Effect of PDT

As a part of non-invasive tumor therapy, PDT has a good application prospect and therapeutic potential. By using a specific laser wavelength to irradiate the photosensitizers and a series of energy and electron conversions, PDT finally produces ROS, which can damage the outer membrane of mitochondria, mediate the mitochondria-related death of tumor cells, and play a therapeutic effect [153]. When the ROS in tumor cells accumulates to a certain level, it will cause lipid peroxidation in tumor cells, then damage mitochondria and endoplasmic reticulum, and finally activate tumor cells death pathway, mediate tumor cells apoptosis, ferroptosis, and other cell death pathways, and achieve the purpose of tumor treatment [154, 155]. Photosensitizer is the core of the therapeutic effect of PDT, which requires good ROS induction ability and high safety under laser irradiation. Curcumin, as a polyphenol compound, can also play the role of photosensitizer under the irradiation of a specific wavelength laser. At the same time, because of its good antitumor ability and high biosafety, it has the potential to be a new photosensitizer [156]. However, the application of curcumin in PDT is limited by its poor water solubility and fast metabolic rate. Curcumin nanoparticles can effectively overcome these shortcomings and mediate an excellent antitumor therapeutic effect [157]. With the development of nanomedicine, curcumin nanoparticles get more functions and achieve better antitumor effects. Jing *et al.* designed FHCPCeNPs with good biocompatibility and ph-responsiveness. Due to the EPR effect, FHCPCeNPs could target tumor cells and then release curcumin and Ce-6 in weakly acidic TME. Under laser irradiation, both curcumin and Ce-6 induced large amounts of ROS to mediate apoptosis in tumor cells. Meanwhile, as a chemotherapeutic agent, curcumin could exert its antitumor effect with PDT [158]. Curcumin nanoparticles with the function of NO self-donor could reduce the oxygen demand rate of tumor cells through NO and alleviate the problem of hypoxia limiting ROS production during curcumin-mediated PDT [159]. Curcumin nanoparticles loaded with MnO_2_ can also effectively alleviate the problem of TME hypoxia, limiting the therapeutic effect of PDT. When the nanoparticles entered the TME, MnO_2_ reacted with H_2_O_2_ in TME under acidic conditions to produce a certain amount of oxygen and improve the production rate of curcumin-induced ROS [160]. Some curcumin nanoparticles can also consume GSH, which could reduce the consumption of ROS induced by curcumin and improve the therapeutic effect of PDT on tumors [161]. In addition, curcumin nanoparticles loaded with chemotherapeutic drugs to achieve PDT combined with chemotherapy can play a synergistic antitumor effect. Curcumin nanoparticles loaded with doxorubicin could be targeted to the tumor cells to achieve targeted delivery of chemotherapeutic drugs. At the same time, curcumin-mediated PDT could induce a large number of ROS and doxorubicin-induced ROS to play a synergistic antitumor effect [162]. The combination therapy can play a synergistic effect and alleviate the problem of chemotherapy resistance to some extent.

Curcumin-mediated PDT can not only treat the tumor itself but also hopefully treat the adverse reactions caused by the tumor treatment. Oral mucosal inflammation during RT or chemotherapy is a common adverse reaction. Some patients will take measures such as freezing and mouthwash to relieve the pain caused by oral mucosal inflammation during treatment [163]. ROS induced by PDT can not only induce tumor cell death but also cause damage to microorganisms [164]. Curcumin can alleviate the side effects of RT and chemotherapy, and it can also be used as a photosensitizer to mediate PDT in treating oral mucosal inflammation in cancer patients. As a photosensitizer, curcumin had an excellent ability to induce ROS and could effectively inhibit the growth of bacteria. The power of curcumin uptake by bacteria and normal cells was different. Curcumin could be effectively enriched in bacteria and play a broad-spectrum antibacterial effect under laser irradiation. At the same time, curcumin-mediated PDT regulated the level of inflammatory cytokines, enhanced the utilization of ATP, promoted the synthesis of fibroblasts and collagen, reduced neutrophil infiltration, and promoted the rapid healing of oral mucosal inflammation caused by radiotherapy or chemotherapy [165, 166]. Due to the limitation of laser tissue penetration, PDT is used chiefly in treating superficial tumors such as breast and skin cancer. However, current studies have confirmed that curcumin-mediated PDT has a certain therapeutic effect on adverse reactions caused by RT and chemotherapy [167]. Curcumin-mediated PDT is expected to be an adjuvant therapy for RT and chemotherapy. It can enhance the antitumor effect, alleviate their side effects, and improve patients' quality of life and prognosis. Moreover, curcumin nanoparticles as photosensitizers can not only solve the shortcomings of curcumin but also solve the potential toxicity of some photosensitizers, especially the potential cardiotoxicity of some photosensitizers. There have been many good results about curcumin-mediated PDT in treating atherosclerosis and other heart diseases. Curcumin, as a photosensitizer, mediates antitumor PDT therapy, and it is a potential research direction to alleviate cardiotoxicity caused by treatment or to assist other therapies.

### 4.5 Curcumin Mediates the Antitumor Effect of SDT

SDT is a new method of tumor treatment in recent years, which is gradually concerning the public because of its non-invasive and safety. SDT refers to the use of a specific frequency of ultrasound irradiation of sonosensitizers through sonoluminescence and sonofever effects to induce a large number of lethal levels of ROS, and then damage mitochondria. With the change of mitochondrial membrane potential and cytochrome C release, tumor cells will die in many ways, such as apoptosis, ferroptosis, *etc* [168, 169]. Different from PDT, ultrasound has more substantial tissue penetration and better therapeutic effect for deep tumors, and the damage of ultrasound to the human body is smaller and safer than laser, which is an essential direction of tumor treatment research in the future. Curcumin, as a photosensitizer, can also play the role of sonosensitizer under ultrasonic irradiation and induce ROS. At the same time, curcumin is a chemotherapeutic drug that can exert the antitumor effect of chemotherapy combined with SDT [170]. Curcumin nanoparticles could effectively solve shortcomings such as poor water solubility of curcumin, and they were constructed together with tumor-derived exocrine, giving the nanoparticles the ability to target tumors. At the same time, loaded CaCO_3_NPs could release calcium ions in acidic TME, cause calcium overload in tumor cells, damage tumor cell mitochondria, and enhance the therapeutic effect of chemotherapy combined with SDT on colon cancer [171]. In addition, studies have shown that curcumin- mediated SDT has a therapeutic impact on liver cancer, oral squamous cell carcinoma, and other tumors, confirming the feasibility of curcumin as a sonosensitizer agent to achieve SDT antitumor [172]. Compared with traditional sonosensitizers, curcumin has higher safety, less biological toxicity, and a safer metabolic pathway, so that curcumin can be widely used in the future.

Cardiotoxicity is a common adverse event in the process of tumor treatment, which affects the treatment regimen, the prognosis of patients, and the quality of life. Curcumin-mediated SDT could promote the apoptosis of macrophages by activating caspase-9, promote the transformation from M1 to M2 reduces the levels of total cholesterol and very low-density lipoprotein and realizes the treatment of atherosclerosis [173, 174]. The anti-inflammatory and antioxidant effects of curcumin can alleviate the cardiotoxicity caused by tumor treatment. Although there is no clear study on the cardiotoxicity induced by curcumin on SDT, it can be speculated that curcumin-mediated SDT can alleviate the cardiotoxicity caused by treatment while achieving antitumor and become the main antitumor treatment or adjuvant therapy. Curcumin, as a sonosensitizer, mediates SDT for antitumor therapy, which is expected to be an effective treatment for tumor therapy and cardiotoxicity caused by therapy in the future.

## 5 CURCUMIN NANOPARTICLES PLAY AN ESSENTIAL ROLE IN NON-INVASIVE ANTITUMOR

Curcumin has various pharmacological effects, such as anti-inflammatory, antioxidation, antitumor, and so on, which determines that curcumin can play an essential role as an antitumor drug or as an antitumor adjuvant in tumor therapy. However, the poor water solubility and rapid metabolism of curcumin limit its clinical application. The construction of curcumin nanoparticles can overcome its shortcomings and promote its application in antitumor. Currently, curcumin nanoparticles mainly include liposomes, nano-micelle, emulsified nanoparticle systems, metal-organic framework-related nanosystems, nonmetal framework-related nanosystems, and so on, which can play a role in various tumor therapy [175-177]. The functional classification of curcumin nanoparticles mainly includes tumor-targeting nanoparticles, ph-responsive nanoparticles, GSH-responsive nanoparticles, relieving hypoxia nanoparticles, **etc*.* Curcumin nanoparticles with more functions can play a better antitumor effect [85, 178, 179]. Loading tumor cell membrane, chitosan, exocrine, GPC3, and other biocompatible substances can improve the biocompatibility of curcumin nanoparticles and have the ability of tumor-targeting, which could effectively play an antitumor role in prostate cancer, liver cancer, and other tumors [180, 181]. Curcumin nanoparticles designed for TME, such as ph-response and GSH-response, could achieve the targeted release in tumor cells of curcumin and other chemotherapeutic drugs and reduce their toxicity to normal tissues. Curcumin nanoparticles with functions of relieving hypoxia and consuming GSH could improve the ability of curcumin to induce ROS and promote ROS- mediated tumor cell death [182, 183]. The application of nanoparticles fosters the possibility of clinical application of curcumin and the combined use of curcumin with other tumor treatments such as RT, chemotherapy, immunotherapy, and so on [184, 185]. Curcumin nanoparticles can play a better antitumor effect, such as increasing the therapeutic effect of other therapies, alleviating side effects caused by treatment, and mediating PDT or SDT in antitumor therapy. Curcumin nanoparticles promote the application of curcumin as a therapeutic adjuvant, alleviate side effects, mediate new tumor treatments, and improve patients' prognosis and quality of life.

## CONCLUSION

As a low-toxic drug extracted from herbaceous plants, curcumin plays a vital role in tumor therapy. Curcumin mainly mediates tumor treatment by inhibiting the NF-κB signal pathway, inhibiting the production of COX-2, reducing the expression of proinflammatory cytokines, and regulating the proportion of immune cells. For some tumor patients who cannot tolerate surgery or cannot be treated by surgery, non-invasive tumor therapy is the first choice for clinically treating tumors. However, the resistance of tumor cells to these treatments and the adverse reactions caused by these treatments, especially cardiotoxicity, seriously affect the quality of life of patients. Diseases such as myocarditis, coronary heart disease, the decline of left ventricular function, pericardial disease, and even heart failure caused by treatment seriously threaten the survival of tumor patients. In addition to its antitumor and adjuvant effects, curcumin can also alleviate adverse reactions in RT, chemotherapy, and immunotherapy. Curcumin has a wide application prospect in antitumor therapy, but its shortcomings limit its application. Curcumin nanoparticles can effectively solve these problems, make it play a better antitumor effect, and alleviate the side effects caused by treatment. At present, most studies have focused on using curcumin as an adjuvant to improve the sensitivity of tumor cells to non-invasive antitumor therapy. However, the alleviating effect of curcumin on adverse reactions caused by therapy, especially cardiotoxicity, in the process of treatment was ignored. Based on the pharmacological action and metabolic kinetic characteristics of curcumin, this paper systematically explains the mechanism of curcumin antitumor, adjuvant therapy, mediating PDT and SDT, alleviating chemotherapy's side effects, and feasible future research direction. As an antitumor drug, curcumin can not only mediate antitumor therapy but also can be used as a therapeutic adjuvant to improve the sensitivity of tumors to other treatments effectively. At the same time, we should pay more attention to its ability to alleviate the pain caused by cardiotoxicity caused by treatment, promote the development of antitumor therapy in a more humane direction, and improve the quality of life of patients.

## Figures and Tables

**Table 1:** The mechanism of curcumin as a chemotherapy adjuvant.

**Chemotherapeutics**	**Mechanism of Action**	**References**
Doxorubicin	Increase DOX absorption, reduce drug efflux, and regulate the intracellular levels of ATP-binding cassette (ABC) drug transporters.	[110]
Cisplatin	Inhibiting PI3K/AKT signaling pathway decreases cisplatin-related toxicity.	[111]
5-fluorouracil	Inhibiting the CAF-induced activation of the JAK/STAT3 signaling pathway and alleviating chemotherapy resistance	[112]
Docetaxel	Enhancing the effect of chemotherapy by promoting the PARP/caspase-3 signaling pathway, up-regulating the TIMP1/TIMP2, and down-regulating the MMP2/MMP9/N-cadherin proteins.	[113]
Oxaliplatin	Inhibiting the expression of CD44 and improving the sensitivity of chemotherapy.	[114]
Metformin	Down-regulating the expression of MMP2/9, VEGF, and VEGFR-2, up-regulating the expression of PTEN, P53, and the suppressing PI3K/Akt/mTOR/NF-κB and EGFR/STAT3 signaling pathway.	[115]
Celecoxib	Increasing cytotoxicity and the activation of caspase-3, reducing the levels of Akt, NF-κB, PGE2, MDA, CD1, and VEGF.	[116]

## LIST OF ABBREVIATIONS

COX-2: Cyclooxygenase-2
RT: Radiotherapy
PDT: Photodynamic Therapy
SDT: Sonodynamic Therapy
MAPK: Mitogen-activated Protein Kinase
LOX: Lipoxygenase
